# Concepts and clinical use of ultra-long basal insulin

**DOI:** 10.1186/s13098-015-0117-1

**Published:** 2016-01-06

**Authors:** Freddy Goldberg Eliaschewitz, Tânia Barreto

**Affiliations:** CPClin Clinical Research Center, 91, Goiás St., São Paulo, SP 01244-030 Brazil; Sanofi´s Medical Division, América Business Park-5200, Major Sylvio de Magalhães Padilha Av., Jd. Morumbi, São Paulo, SP 05693-000 Brazil

**Keywords:** Diabetes mellitus, Ultra-long basal insulin, Glycemic control, Hypoglycemia

## Abstract

Diabetes mellitus (DM) is a public health issue, affecting around 382 million people worldwide. In order to achieve glycemic goals, insulin therapy is the frontline therapy for type 1 DM patients; for patients with type 2 DM, use of insulin therapy is an option as initial or add-on therapy for those not achieving glycemic control. Despite insulin therapy developments seen in the last decades, several barriers remain for insulin initiation and optimal maintenance in clinical practice. Fear of hypoglycemia, weight gain, pain associated with blood testing and injection-related pain are the most cited reasons for not starting insulin therapy. However, new generation of basal insulin formulations, with longer length of action, have shown the capability of providing adequate glycemic control with lower risk of hypoglycemia.

## Insulin therapy development

Diabetes mellitus (DM) affects around 382 million people worldwide, with type 2 DM accounting for 85–95 % of all cases [1]. Diabetes may be managed with tight glycemic control, aiming to achieve glycosylated hemoglobin (A1C) levels below or around 7 %, considering even more stringent goals (such as <6.5 %) for selected patients. This can be achieved without significant adverse events, as hypoglycemia [2], due to the relation of lower A1C levels with fewer microvascular complications [3–6]. Insulin therapy is recommended as frontline therapy for type 1 DM patients; for patients with type 2 DM, use of insulin is an option as initial or add-on therapy for those not achieving glycemic control with initial oral drugs [2]. Currently, only in the United States, around 6 million people aged ≥18 years with DM use insulin therapy (28.7 % of the DM patients population in this age in the country) [7].

Insulin was first used in diabetic patients in the 1920s [8], but the first commercial preparations contained numerous impurities and relevant potency variation [9]. In the 1930s protamine zinc insulin was developed, resulting in delayed absorption and longer duration of action, thus reducing the number of doses needed for insulin replacement—but still with considerable instability [10]. In 1946, neutral protamine Hagedorn (NPH) insulin was produced and could be pre-mixed with soluble insulin, becoming the main basal insulin throughout the 20th century [10].

In the early 1980s, the development of human insulin innovated with refinement of insulin therapy, especially regarding basal insulin [9]. New long-acting insulin analogues, glargine and detemir, showed less day-to-day variability and longer duration of action, thus allowing once-daily dosing [11, 12].

**Fig. 1:**
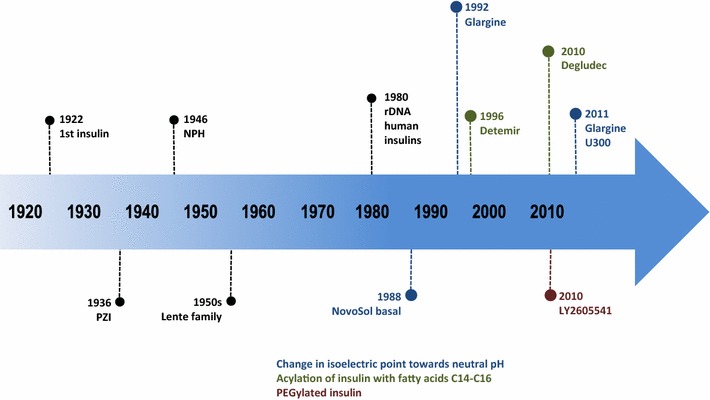
Timeline for insulin developments. Adapted from Owens [10]

The search for an ideal insulin therapy regimen still continues, aiming to provide optimal glycemic control with limited adverse events and improved convenience to the patient [13] (Fig. 1).

## Pharmacokinetic and pharmacodynamics profiles of insulin therapy

Overall development of insulin aims to decrease patients’ hypoglycemia episodes and improve pharmacokinetics (PK)/pharmacodynamics (PD) profile, by mimicking as close as possible the normal insulin release [14]. Variability of PK profile among insulins directly influences PD effects, increasing the probability of hypoglycemia episodes due to unpredictability of insulin peaks. In addition, temporal separation of insulin PK and PD profiles is affected by several factors, thus PK and PD profiles of insulin may be studied separately, each with its specific measurement [15, 16].

**Fig. 2:**
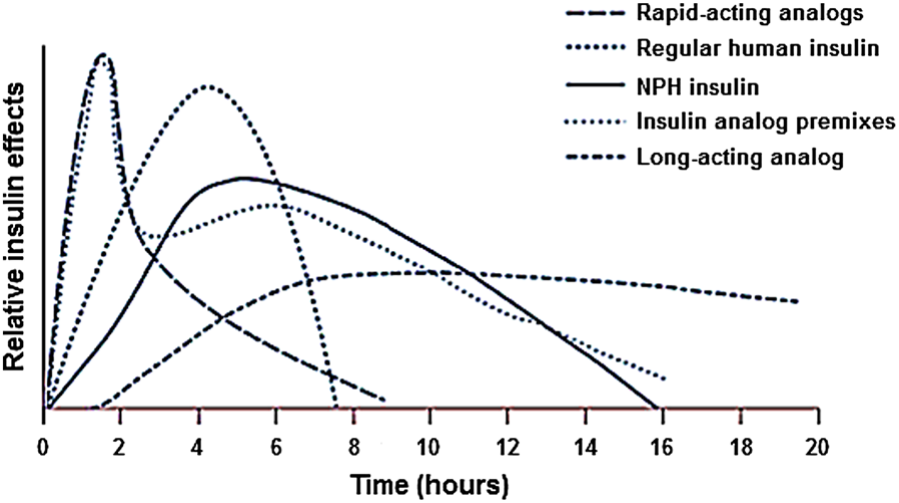
Comparison between the action time profiles of different insulins. Adapted from: Tibaldi [59]

In order to minimize hypoglycemia or hyperglycemia episodes, insulin analogues with different PK/PD profiles were developed, including basal and ultra-long basal insulins. Unfortunately, current insulin options do not fulfill the main requirements for ideal basal insulin: flat PD profile, with low hypoglycemia risk, 24 h duration, and low interindividual variability (Fig. 2).

Insulin NPH has a delayed absorption profile, with a peak in 4.5 h after administration. Besides, insulin NPH action doesn’t last for 24 h, so usually it has to be administered at least twice a day. Furthermore, NPH treatment has high inter and intraindividual variability [17]. Thus, other insulins were developed to improve PK/PD profile.

In 2000, insulin glargine was the first long-acting insulin analogue available. After subcutaneous injection, glargine precipitates in the subcutaneous tissue delaying the absorption [18]. Insulin glargine has one amino acid substituted in the A chain and two in the B chain: this modifies the isoelectric point of the protein, decreasing its solubility at physiologic pH. Due to its delayed absorption, glargine also demonstrates a flatter PK/PD profile, with longer duration and less variability when compared to NPH [17].

Insulin detemir is another bioengineered insulin developed by removing a threonine and acylating a lysine residue with 14-carbon fatty acid, both in the B chain. It has a PK/PD profile very similar to glargine (flatter PK/PD profile, approximately 24-h duration and low variability), although insulin glargine is slightly more effective and stable in glycemic control [19].

However, both glargine and detemir still do not completely mimic physiological insulin secretion. When administered in high doses, both show a peak on PK/PD profile [20]; also, a low dose may not be enough to cover a 24-h period and there is still interindividual variability [21]. To attempt to overcome this issue, ultra-long insulins were developed, being degludec, LY2605541 and glargine U300 the most promising.

Insulin degludec is a modified B chain analogue that, like other insulin, forms hexamers and di-hexamers when injected subcutaneously. As the slow diffusion of zinc molecule, present in the hexamer, occurs, the insulin monomer is finally absorbed. Compared with insulin glargine and detemir, degludec promotes flatter PK/PD profiles, decreasing the number of confirmed hypoglycemia episodes [22]. Insulin degludec has a half-life superior to 25 h, with action exceeding 42 h, however the most studied schedule in clinical trials is based on once daily doses.

Insulin LY2605541 (or PEGylated Lispro) has polyethylene glycol (PEG) polymer chain attached in the molecule structure. PEGylation slows LY2605541 subcutaneous absorption and decrease its renal clearance, promoting the creation of a peakless PK/PD profile and increasing LY2605541 half-life. LY2605541 presents a PK/PD profile very similar to insulin degludec, with mean half-life of 35 h [23, 24] but, differently from degludec, LY2605541 causes weight loss and hepatic lipid accumulation in both type 1 [25] and type 2 DM [24]. The mechanisms of hepatic lipid accumulation are unknown but led to the discontinuation of this insulin.

**Fig. 3:**
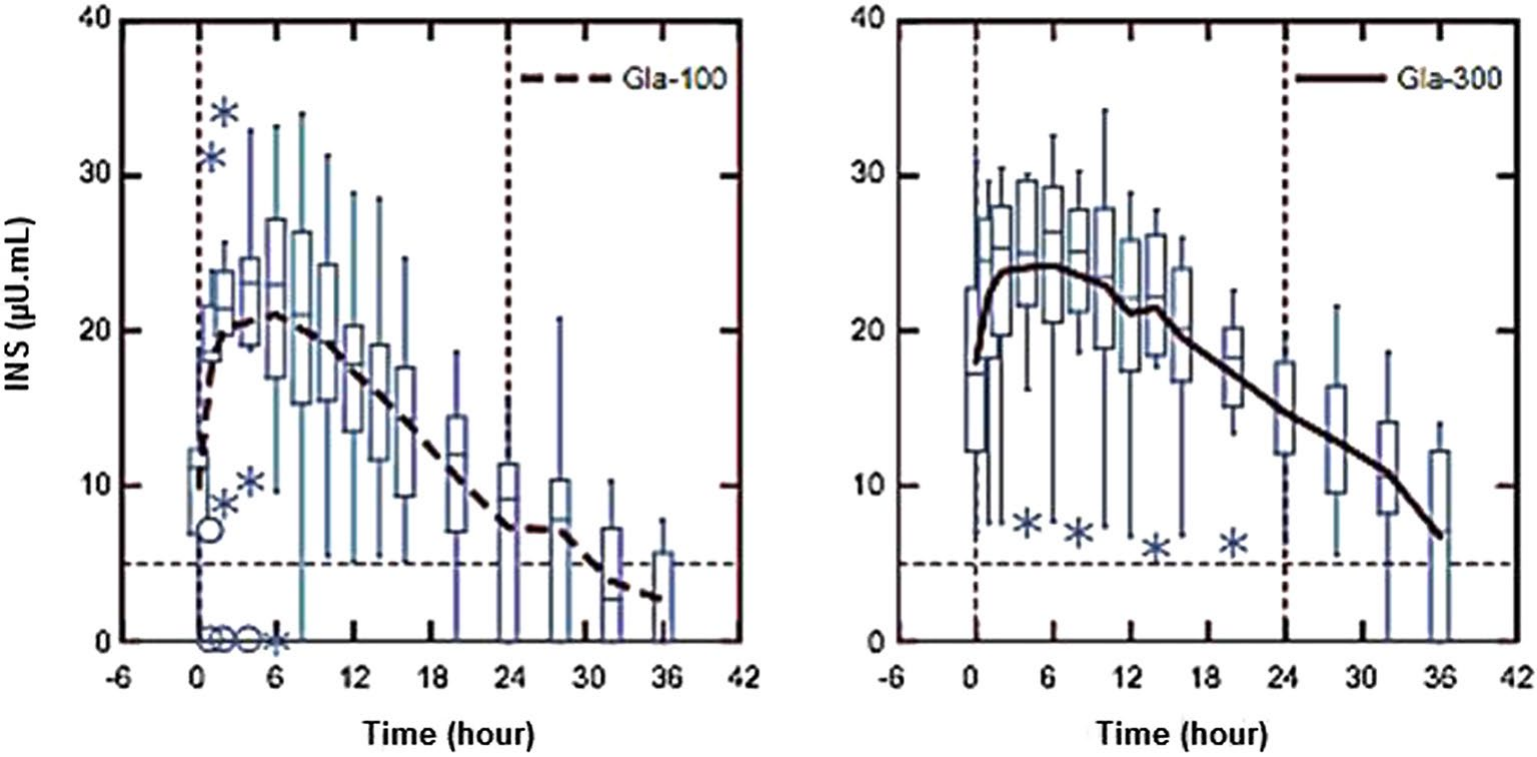
Concentration of insulin glargine U300 versus U100 over time in steady. Adapted from Becker et al. [26]

The most recent clinical approach for ultra-long insulin is glargine U300 (300 U/ml). The analogue has the same mechanism to extent absorption as insulin glargine. When administered subcutaneously, U300 forms subcutaneous depot with smaller surface area, creating a prolonged release that results in a flatter PK/PD profile than in glargine U100. Furthermore, exposure to U300 is more evenly distributed (Fig. 3) [26], and glucose control remains for 36 h [27], resulting in decreased hypoglycemic episodes (overall and nocturnal) in insulin-based [28] or insulin combined with oral antihyperglycemic drugs therapy [29]. U300 also demonstrated reduced variability and a tendency to decrease weight gain compared with glargine U100.

It should be highlighted that insulin therapy should always try to mimic the physiological insulin release, thus longer half-life insulin is not always the best choice. As an example, ultralente insulin has a mean duration of 20 h, which is superior to 14 h of insulin NPH [17]. However, this insulin presents higher interindividual variability, higher incidence of hypoglycemia episodes and worse glucose control when compared with NPH [17, 26]. Due to its inferior clinical profile, ultralente was withdrawn from the market in 2005.

Ultra-long insulins offer efficacy and safety, with less pharmacodynamics variability and intraindividual variability, and duration of action over 24 h. Among these insulins, both U300 and degludec can be used as basal and basal-bolus therapy, in type 1 and type 2 DM, offering the advantage of flexibility. This means that healthcare professionals and diabetic patients don’t need to be rigid about the timing of injections.

## Clinical trials evaluating ultra-long-acting insulin formulations

### Insulin degludec for type 1 DM

BEGIN was an open-label, treat-to-target, non-inferiority, multicentric trial assessing adult patients with type 1 DM who had been treated with basal-bolus insulin for at least 1 year (70 % receiving insulin glargine and 19 % receiving insulin detemir at screening) [23]. In total, 626 participants were randomized in a 3:1 ratio to receive once-daily insulin degludec (100 U/mL; 3 mL) or insulin glargine (100 U/mL; 3 mL), both in combination with mealtime insulin aspart. Primary objective was to confirm non-inferiority of degludec over glargine in reducing A1C levels after 52 weeks of treatment [23]. Insulin degludec demonstrated to be non-inferior, with a reduction in A1C levels by 0.4 % points versus 0.39 % points with glargine (difference of −0.01 % points; 95 % CI −0.14–0.11; p < 0.0001 for non-inferiority). Rates of overall confirmed hypoglycemia were similar in both groups, but the rate of confirmed nocturnal hypoglycemia was 25 % lower with degludec (4.41 versus 5.86 episodes/patient-year of exposure; relative risk 0.75; CI 95 % 0.59–0.96). No differences in weight gain or in other adverse events were observed between study groups [23]. A 52-week extension of the BEGIN trial corroborated the benefits of degludec on confirmed nocturnal hypoglycemia, with a sustained event reduction of 25 % (CI 95 % from 5 to 41 %), without significant differences in efficacy or in other adverse events [30]. These data evidence the efficacy and safety of degludec, as well as its non-inferiority in relation to insulin glargine.

A subsequent trial (BEGIN: Flex T1) investigated efficacy and safety of insulin degludec once daily, but varying the injection time [31]. This was an open-label, treat-to-target, non-inferiority trial comparing insulin degludec (100 U/mL) in a fixed schedule (with minimum 8 h and maximum 40 h between doses) with degludec (100 U/mL; 3 mL) or glargine (100 U/mL; 3 mL) given at the same time every day, once daily, with mealtime insulin aspart. After 26 weeks of treatment, mean A1C and fasting plasma glucose levels were reduced similarly in the 3 groups. Insulin degludec in the flexible schedule achieved non-inferiority and significantly reduced confirmed nocturnal hypoglycemia [31]. Also, the study highlighted the advantage of the long duration of degludec in offering more flexibility in terms of injection timings.

### Insulin degludec for type 2 DM

BEGIN basal-bolus type 2 study was an open-label treat-to-target, non-inferiority trial, performed at 123 sites in 12 countries. The study enrolled adult patients with type 2 DM who had been treated with any insulin regimen for at least 3 months with or without oral antidiabetic drugs and had A1C concentrations of 7.0–10.0 % [32]. This trial randomized 1006 patients (3:1) to receive once-daily insulin degludec (100 U/mL; 3 mL) or glargine (100 U/mL; 3 mL), in combination with mealtime insulin aspart, with or without prescribed metformin, pioglitazone or both. The primary objective was to confirm the non-inferiority of degludec over glargine in reducing A1C levels. After 1 year, A1C decreased by 1.1 % in the degludec group and 1.2 % in the glargine group (estimated difference degludec-glargine: 0.08 %; 95 % CI −0.05 to 0.21), confirming its non-inferiority [32]. Rates of overall confirmed hypoglycemia were lower with degludec (11.2 versus 13.6 episodes/patient-year of exposure; estimated ratio 0.82; 95 % CI 0.69–0.99), as well as rates of confirmed nocturnal hypoglycemia (1.4 versus 1.8 episodes/patient-year of exposure; estimated ratio 0.75; 95 % CI 0.58–0.99). Rates of severe hypoglycemia were too low to assess differences, and adverse events occurred in a similar rate in both groups [32]. A 26-week extension showed that degludec maintained similar improvement in glycemic control compared with glargine, with fewer hypoglycemic episodes (24 % overall reduction and 31 % confirmed nocturnal episodes reduction) [33].

Another 1-year large phase 3 trial (BEGIN Once Long) randomized 1030 patients (3:1) with type 2 DM, insulin-naïve, inadequately controlled with oral antidiabetic drugs (A1C 7–10 %) to receive insulin degludec or glargine once daily, both combined with metformin [34]. Insulin degludec showed similar rates of glycemic control but, again, with reduced risk of confirmed nocturnal hypoglycemia as well as of severe adverse events [34]. The 52-week extension of BEGIN Once Long trial showed sustained benefits of insulin degludec in hypoglycemic episodes [35].

Flexible schedule of administration of insulin degludec has also been studied in type 2 DM patients. BEGIN Flex trial assessed 687 patients (41.8 % already receiving insulin therapy) randomized to receive degludec in a flexible once-daily schedule (with prespecified rotating doses of 8–40 h intervals), degludec once daily at the main evening meal, or glargine [36]. In this trial, no differences between groups were reported on glycemic control, hypoglycemia or adverse events, suggesting that daily injection time of insulin degludec can vary without compromising efficacy [36].

### Insulin glargine 300 units/mL for type 1 DM

EDITION 4 trial is a randomized, phase 3a, open-label study recently published (2015). The study randomized 549 patients (1:1:1:1) to receive once-daily insulin glargine 300U or 100U, in the morning or evening, while continuing mealtime insulin. Participants had diabetes duration of 21 years and HbA1c level of 8.1 % [37]. Overall, glargine 300U was non-inferior over glargine 100U for the primary endpoint (HbA1c change from baseline), with reductions of 0.40 versus 0.44 %, respectively (mean difference 0.04; CI 95 % −0.10 to 0.19 %). Event rates of confirmed or severe hypoglycemia (≤3.9 mmol/L) were similar in both groups while nocturnal hypoglycemia was lower in glargine 300U group during the first 8 weeks of the study [37]. Insulins or times of injection showed no differences in glycemic control, hypoglycemia or adverse events between groups. Glargine 300U led to a statistically significant lower weight gain (mean difference −0.56 kg; CI 95 % −1.09 to −0.03 kg) [37]. Also, the convenience of a once-daily injection and lower rates of hypoglycemia should convert into improvements in treatment satisfaction.

### Insulin glargine 300 units/mL for type 2 DM

EDITION 1 was a 6-month multinational (conducted in 13 countries), open-label trial that assessed adult patients with type 2 DM who were receiving basal-bolus insulin therapy (requiring ≥42 units/day of basal insulin) for at least one year (57.4 % with prior use of metformin; other oral antidiabetic therapies were not allowed) and with A1C levels 7.0–10.0 %. Participants (n = 807) were randomized to receive once-daily injections of insulin glargine 300U or 100U through 6 months [28]. Reduction in A1C levels (primary endpoint) was similar in both groups (mean change of −0.83 % for both groups), meeting the non-inferiority criterion. The main secondary endpoint was the percentage of patients with one or more confirmed or severe nocturnal hypoglycemia (≤3.9 mmol/L) reported between week 9 and month 6 and patients receiving glargine 300U achieved better results, with a 21 % risk reduction (relative risk 0.79; 95 % CI 0.67–0.93). Adverse events or treatment-emergent adverse events were equally distributed between the groups [28]. A pre-planned 6-month extension showed sustained glycemic control and less hypoglycemia with glargine 300U [38]. A lower percentage of participants receiving insulin glargine 300U experienced confirmed or severe hypoglycemia (85.9 versus 91.5 %; RR = 0.94; CI 95 % 0.89–0.97) and the annualized rate of documented symptomatic nocturnal hypoglycemia was lower in this group (1.8 versus 2.5 per participant-year; RR = 0.74; CI 95 % 0.56–0.97) [38].

EDITION 2 was a randomized phase 3 trial with a design similar to EDITION 1: it was a 6-month, open-label trial conducted in 213 centers across 13 countries, analyzing adult patients with type 2 DM who were receiving basal insulin therapy (requiring ≥42 units/day of basal insulin) for at least 1 year and with A1C levels 7.0–10.0 % [29]. This study differed from EDITION 1 because patients were not receiving mealtime rapid-acting insulin and they could receive other antidiabetic therapies, except sulphonylureas and glinides (in 94 %, oral therapy was metformin). Participants (n = 811) were randomized to receive once-daily injections of insulin glargine 300U or 100U in the study period. Primary endpoint was change in A1C levels from baseline to month 6 and the results were statistically similar in both groups (mean change of −0.57 % for glargine 300U and −0.56 % for glargine 100U), meeting the non-inferiority criterion. The main secondary endpoint was the percentage of patients with one or more confirmed or severe nocturnal hypoglycemia event (≤3.9 mmol/L) reported between week 9 and month 6 and it was lower in patients receiving glargine 300U (21.6 %) than glargine 100U (27.9 %), with a risk reduction of 23 % (relative risk of 0.77; 95 % CI 0.61–0.99). EDITION 2 participants continued in a 6-month safety extension in order to examine long-term outcomes [39]. Over 12 months, improved control of HbA1c was maintained in both treatment groups, and event rates/participant-year of confirmed or severe nocturnal hypoglycemia were 37 % lower with insulin glargine 300U (1.74 versus 2.77; RR = 0.63; CI 95 % 0.42–0.96) [39].

EDITION 3 is another multicentric randomized trial evaluating insulin glargine 300U, but assessing insulin-naive adult patients with type 2 DM on oral glucose-lowering drugs [40]. Participants (n = 878) were randomized to receive once-daily injections of insulin glargine 300U or 100U, throughout the 6-month study period, after discontinuing sulphonylureas and glinides. The primary endpoint was change in A1C levels from baseline to month 6 and the results showed an equivalent reduction in both groups (mean change of −1.42 % for glargine 300U and −1.46 % for glargine 100U; mean difference 0.04 %; CI 95 % −0.09 to 0.17 %), meeting the non-inferiority criterion. In this trial, non-inferiority was pre-defined as a difference <0.4 % in A1C levels between the treatment groups. The main secondary endpoint was the percentage of patients with one or more confirmed or severe nocturnal hypoglycemia event (≤3.9 mmol/L) reported between week 9 and month 6 and it was statistically similar in patients receiving glargine 300U (16 %) or glargine 100U (17 %). When considering the whole 6-month treatment period, fewer patients receiving glargine 300U experienced such events (18 versus 24 %; RR = 0.76; CI 95 % 0.59–0.99) [40].

Flexible dosing intervals of insulin glargine 300U were also tested in type 2 DM patients. Patients participating in EDITION 1 (n = 109) and EDITION 2 (n = 89) using glargine 300U were randomized, at month 6, to continue the fixed regimen or move to flexible regimen, allowing between-injection intervals of 24 ± 3 h on at least 2 days each week [41]. In the latter group, throughout this 3-month flexibility sub-study, only 50–60 % of injections ranged 24 ± 1 h. HbA1c change was comparable in fixed versus flexible regimens and hypoglycemia events occurred equally between both groups, showing that glargine 300U provides flexibility to occasionally adapt the timing of insulin injections to individuals’ daily changing lifestyle patterns [41].

## Barriers for insulin therapy implementation and unmet needs: emphasis in hypoglycemia

Several barriers remain for insulin initiation and optimal maintenance in clinical practice, both at the patient and at the physician levels. Fear of hypoglycemia, weight gain, pain associated with blood testing and injection-related pain are the most cited reasons for not starting insulin therapy in type 2 DM patients [42]. Also, patient perception that insulin therapy is complicated and time consuming can interfere with its timely initiation [43]. Adherence to insulin therapy, however, is more influenced by factors like health insurance patterns and being too busy [44, 45].

Fear of hypoglycemia impacts not only in the decision to start insulin therapy, but it may also jeopardize the adequacy of glycemic control. It has been reported that most physicians would treat patients without adequate glucose control in a more aggressively manner if not for concerns about hypoglycemia [45].

## Burden of hypoglycemia in patients with dm receiving insulin therapy

Hypoglycemia is an important adverse event of DM therapy as it potentially impairs the patients’ health-related quality of life while imposing other burdens [17, 18]. Observational studies report that hypoglycemia occurs in up to 42.89 events/patient-year, in type 1 DM patients, and in up to 16.36 events/patient-year in insulin-treated type 2 DM patients [46, 47]. Rates of severe hypoglycemia are approximately of 1.15 events/patient-year, and can reach 3.2 events/patient-year in type 1 DM patients; in insulin-treated type 2 DM patients, rates are 0.7 events/patient-year [46, 47].

Nocturnal hypoglycemia, which occurs during sleep, is particularly dangerous since patients are unlikely to recognize symptoms or awake during an episode [48]. In the Diabetes Control and Complications Trial (DCCT), 43 and 55 % of all hypoglycemic and severe hypoglycemic events reported, respectively, occurred during sleep [49].

A large web survey conducted in the US with 7.239 participants with type 2 DM (28.7 % treated with insulin) showed that hypoglycemia interfered with social activities, causing more absenteeism and decreasing overall work productivity, also impacting negatively on overall health-related quality of life [50]. Another survey, performed in US and Europe, attested that even non-severe hypoglycemic events cause major impairments on productivity, with productivity loss estimated from U$ 15.26 to U$ 93.47, representing 8.3 to 15.9 h of lost work time per month [51]. Also, a time trade-off utility study with DM patients receiving insulin therapy in US and Canada showed that non-severe hypoglycemic episodes led to deterioration in utility [52]. Further research has reported that patients with confirmed hypoglycemia episodes had significant impairment in health-related quality of life [53], greater mood disturbance and less work satisfaction [54].

Specific burden of nocturnal hypoglycemia has also been studied. A survey recruited DM patients in US, Canada and in 7 European countries who experienced a non-severe nocturnal hypoglycemia event in the previous month [55]. The 2108 respondents (32.8 % with type 1 and 67.2 % with type 2 DM; 74.2 % receiving insulin therapy) reported that hypoglycemic episodes significantly affected sleep and next day functioning, with 60.7 % stating a moderate to severe impact [55]. Another survey assessing 8286 patients in 5 countries (US, Canada, Germany, Sweden and United Kingdom) presented that living in a health state with nocturnal hypoglycemia is considered worse than living with daytime hypoglycemia [56]. Other studies also reported the detrimental impact of nocturnal hypoglycemia on quality of life, including impact on family members and cost-related factors [57].

The potential association of hypoglycemia with neurological impairment is also worrisome. A longitudinal study following 16,667 older patients with type 2 DM (mean age: 65 years; 35 % receiving insulin therapy) through 27 years showed an increased risk in dementia among patients with reported hypoglycemic episodes, with risks growing as the number of episodes increased [58].

Additionally, hypoglycemic episodes can lead patients to exhibit medication-avoidance behavior, with many reducing or even omitting insulin doses after an event, which may jeopardize glycemic control [51, 55, 57].

## Conclusion

Patients with DM benefit from a tight glycemic control strategy, which requires insulin therapy for type 1 DM as well as for many patients with type 2 DM. Achieving adequate target levels of blood glucose, however, may lead to higher incidence of hypoglycemic episodes, including nocturnal events, which are associated with impairment in productivity and in health-related quality of life, and may also jeopardize cognition in older patients. Furthermore, hypoglycemia is considered a barrier for implementing more intensive therapies, which can reduce the chances of attaining optimal glucose control.

New generation of basal insulin formulations, with longer length of action, showed the capability of providing adequate glycemic control, in the same extent as insulin glargine, but with decreased risk of hypoglycemia, especially nocturnal episodes. Thus, they help fulfilling the need to adequate glycemic control with lower risks of hypoglycemic events.

The scientific grounds regarding ultra-long basal insulins provide possibilities of its adoption either as upfront therapy as well as, in scenarios with cost constraints, for patients already receiving long-acting insulins glargine or detemir but that have suffered from recurrent episodes of hypoglycemia.

## Abbreviations

CI: confidence interval
DM: diabetes mellitus
GIR: glucose infusion rate
Gla-100: insulin glargine 100 U
Gla-300: insulin glargine 300 U
NPH: neutral protamine Hagedorn
PD: pharmacodynamics
PK: pharmacokinetics
RR: relative risk
T1DM: type 1 diabetes mellitus
T2DM: type 2 diabetes mellitus
